# Synergistic Enhancement of the Friction and Wear Performance for UHMWPE Composites under Different Aging Times

**DOI:** 10.3390/polym16142059

**Published:** 2024-07-19

**Authors:** Yingliang Liu, Yunxiang Han, Lin Yuan, Jinming Zhen, Zhengfeng Jia, Ran Zhang

**Affiliations:** College of Materials Science and Engineering, Liaocheng University, Liaocheng 252059, China; m19861208278@163.com (Y.L.); 19111912571@163.com (L.Y.); jiazhengfeng@lcu.edu.cn (Z.J.); zhangranlicp@163.com (R.Z.)

**Keywords:** UHMWPE matrix composite, dry sliding, ceramic particle, aging time

## Abstract

With the rapid development of the pipeline transportation and exploitation of mineral resources, there is an urgent requirement for high-performance polymer matrix composites with low friction and wear, especially under oxidative and prolonged working conditions. In this work, ultra-high-molecular-weight polyethylene (UHMWPE) matrix composites with the addition of carbon fibers (CFs), TiC, and MoS_2_ were prepared by the hot press sintering method. The influence of thermal oxygen aging time (90 °C, 0 h–64 h) on their mechanical and frictional performance was investigated. The results showed that TiC ceramic particles can increase wear resistance, especially by aging times up to 32 and 64 h. The wear mechanisms were analyzed based on the results of SEM images, EDS, and Raman spectra. The knowledge obtained herein will facilitate the design of long-service-life polymer matrix composites with promising low friction and wear performances.

## 1. Introduction

Due to its low friction, excellent wear resistance, and physical/chemical properties, UHMWPE is one of the most remarkable materials offering promising applications in the biomedical and mechanical industries [1,2,3,4]. However, during operation, high friction and wear of service materials occur when starting and stopping UHMWPE devices, so high-performance composites with excellent self-lubricating properties are urgently needed to prolong service lives. For pure polymer materials, low hardness, poor thermal conductivity, easy creep/entangle, poor processing properties, etc. are all problems encountered when they are worked with [5,6,7,8]; therefore, both the mechanical and frictional properties of these composites need to improve.

As one of the most commonly used modification methods, filled modification has had many studies carried out by researchers; most parts of the used substances are filled and modified with organic particles, inorganic particles, polymers, etc. [9,10,11,12,13]. Cai et al. [14] reported that organically modified α-zirconium phosphate (CZrP) gradually accumulates on the worn surface during the process of dry sliding friction and wear and forms a protective layer with a certain strength and lubricity through the unique interlayer sliding effect, thereby reducing the friction coefficients and wear rates of CZrP/UHMWPE composites. Wang et al. [15] investigated the frictional performance of hybrid attapulgite (ATP) loaded with titanium dioxide (TiO_2_)-modified UHMWPE matrix composites and found that the filler (ATP-TiO_2_) promoted transfer film formation and prevented the severing of the worn surface and that the optimal content was 5 wt. %. By adding hybrid graphene nanoplatelets (GNs) and titanium nitride (TiN) nanoparticles, Uyor et al. [16] prepared UHMWPE nanocomposites by solvent mixing/the hot pressed sintering method and studied the wear properties for these composites; scanning electron microscope (SEM) images presented that the nanoparticles’ uniform dispersion and the wear resistance/thermal stability performances were quite improved compared to pure UHMWPE due to the network structures of the GNs and TiN nanoparticles formed in the UHMWPE matrix. Dong et al. [17] prepared a new thermoplastic polyurethane (TPU)/boron nitride (h-BN) composite material; their frictional vibration experiment showed that the addition of h-BN gave TPU material excellent self-lubricating properties and significantly reduced the wear rate. To improve the tribological performance of poly-ether-ether-ketone (PEEK), Zhao et al. [18] investigated different types of fillers and polymers in a hybrid PEEK-polytetrafluoroethylene (PTFE)-steel wear system; the results showed that the improved wear resistance properties strongly depend on the types of fillers and that the synergistic effect between PEEK and PTFE could improve tribological performance. Wang et al. [19] prepared a PTFE/glass fiber composite and investigated the effects of test temperature and vacuum degree on the frictional behavior of the materials; the results showed that abrasion and fatigue wear are the main wear mechanisms in a vacuum environment of 30 to −60 °C when compared to those under atmospheric conditions. In order to improve the mechanical and wear resistance properties for UHMWPE materials, Zhang et al. [20] prepared UHMWPE/carbon nanotube composites, and the friction test showed that the friction coefficient and wear rate were significantly reduced due to the contribution of the nano-sized filler. Gürgen et al. also [21] introduced the nano fumed silica into a UHMWPE matrix and prepared the composites; wear tests presented that the addition of fumed silica could enhance anti-oxidation behavior and decrease the wear rate under low load; excessive loading led to the formation of particle clusters.

Apart from adding solid lubricant and hardener to improve the friction and wear behavior of composite materials, it is also quite important for researchers to study the environment (like the aging time, testing temperature and lubricating environment, etc.) intensively as the frictional process is very complicated [18,22,23,24,25,26]. Lin et al. [27] investigated the effect of sliding speed on the friction and wear behavior of lubricant-reinforced 304 stainless steel composites with UHMWPE coating; the results showed that PTFE-reinforced composites exhibited the best friction reduction and that the optimum sliding speed was below 200 rpm. Guo et al. [28] investigated the tribological properties of microcapsules/UHMWPE composites under a water environment; the experimental results presented that the incorporation of microcapsules could optimize surface morphology and further reduce the friction coefficient and wear rate. Qin et al. [9] developed a new nitrile rubber containing UHMWPE and graphite and found that it could reduce the coefficient of friction at low speeds and meet the requirements of marine standards. Zhang et al. [29] investigated the effects of carbon fiber fillers on the mechanical and tribological properties of polyamide6/polyphenylene sulfide (PA6/PPS) composites and found that the addition of carbon can improve the lubricating performance while reducing wear-resistant properties. Moreover, surface treatment also had an important effect on the frictional behavior of composites [30]. Shao et al. [31] studied the influencing mechanism of laser treatment on a UHMWPE composite’s tribological performance under sea water; the results showed that oxidation and amorphization decrease the wear resistance properties while carbonization significantly decreases the wear rate and friction coefficient. Using a pin-on-disk tribotester, Menezes et al. [32] investigated the transfer film formation during the sliding process occurring between UHMWPE pins and steel plates; the testing results showed that the surface texture had an effect on the friction coefficient and that the transfer film that formed on the sliding surface was dependent on these values. Sanchez et al. [33] studied the tribological behavior of micro-textured UHMWPE-AISI316L tribopair and the results showed that since the applied loads were not enough to produce a large contact area, the friction coefficient was higher than that of a smooth surface under the distilled water condition.

As mentioned above, the research on inorganic fillers modified with UHMWPE composite materials has received extensive attention. Moreover, in our previous work, we studied the influence of aging time on the friction and wear behavior of UHMWPE composites and found that MoS_2_ and CF have an excellent synergistic lubricating effect [25]. In order to further improve the friction behavior of UHMWPE under oxidative conditions, the UHMWPE matrix composites with CF, MoS_2_, and TiC ceramic particles were prepared by the hot press sintering method, the influence of aging time on mechanical/tribological behavior was systematically investigated, and the corresponding wear mechanisms were systematically analyzed.

## 2. Experimental Section

### 2.1. Material Preparation

The UHMWPE matrix composites were first mixed by ball milling at a rotational speed of 300 rpm for 2 h. Then, the mixed materials were put into a stainless steel die and placed in a hot press sintering furnace. The compositions for the composite materials are shown in Table 1. Powders of MoS_2_ (AR, 99%, Shanghai McLean Biology Co., Ltd., Shanghai, China), TiC (Shanghai Naiou Nano Technology Co., Ltd., Shanghai China), CF (Jilin Chemical Fiber Co., Ltd., Jilin, China), and UHMWPE (GUR4120) were used in this work. Thermal aging was conducted in a 401A hot air aging test chamber (Qidong Shuangleng Test Equipment Co., Ltd., Qidong, China) for different times from 0 to 64 h and the thermal aging temperature in this study was set as 90 °C.

### 2.2. Tribological Performance Tests

The dry sliding wear tests of the UHMWPE matrix composites were performed using a ball-on-disk tribotester (HT-1000, Lanzhou Zhongke Kaihua Technology Development Co., Ltd., Lanzhou, China). The coefficient of friction (COF) data were collected in real time by computer and the detailed tribotest parameters are shown in Table 2. To ensure the accuracy of the data and reduce error, all friction tests were repeated at least three times under the same test conditions and the average value was taken as the final COF. After the reciprocating friction test, a contact probe (MT-500, Lanzhou Zhongke Kaihua Technology Development Co., Ltd., Lanzhou, China) was used to measure the wear volume. Then, the wear volume (mm^3^) was divided by load (N) and sliding distance (m), and after that, the wear rate was obtained and expressed in mm^3^/Nm. Moreover, the 3D topographies of the worn surface were recorded using a white light interferometer (SuperView W3, Shenzhen Chotest Technology Development Co., Ltd., Shenzhen, China).

### 2.3. Characterization

The mechanical property tests were performed on an electronic universal material testing machine with a 20 kN load cell (MTS, E44.304, 20 kN). The phase composition and thermal stability of the UHMWPE composites were characterized using X-ray diffraction (XRD, TD-3700, Liaoning, China), differential scanning calorimetry analysis (DSC, FTA, 449C, Netzsch, Selb, Germany), and thermogravimetric analysis (TGA, Mettler Toledo, Greifensee, Switzerland). To further investigate the wear mechanism, scanning electron microscope (FE-SEM, LSM5600LV) and Raman spectrum (Renishaw inVia Reflex, with a wavelength of 532 nm) were performed to observe the morphology and composition of the sliding surface.

## 3. Results and Discussion

**Figure 1:**
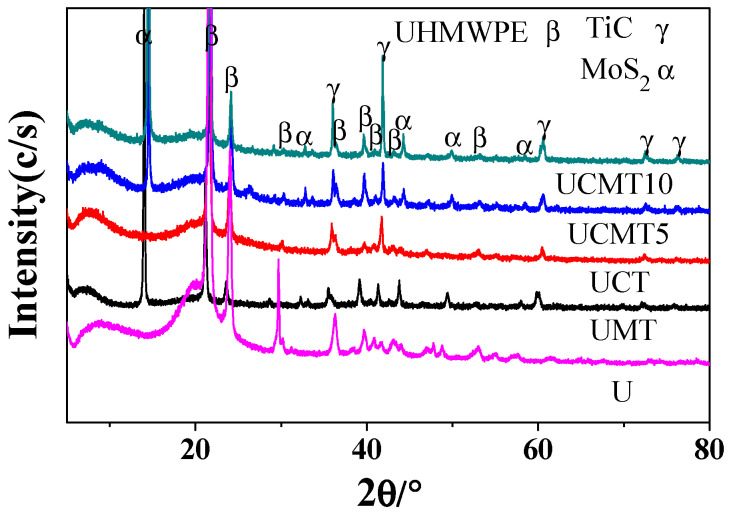
XRD pattern of the UHMWPE matrix composite.

To characterize the composition of the composites, an XRD pattern was created as shown in Figure 1. The composites consisted of three types of phases: a UHMWPE matrix (three diffraction peaks at 2θ of 21.4°, 24.1°, and 36.2°), ceramic particles (TiC) (five diffraction peaks at 2θ of 35.9°, 41.7°, 60.5°, 72.4°, and 76.2°) and solid lubricants (MoS_2_) (six diffraction peaks at 2θ of 14.4°, 32.7°, 39.5°, 44.1°, 49.8°, and 58.3°), which were consistent with the standard data. Moreover, it can also be clearly seen in the figure that the relative intensity of the matrix decreased with an increasing MoS_2_ content, which was consistent with the original design.

**Figure 2:**
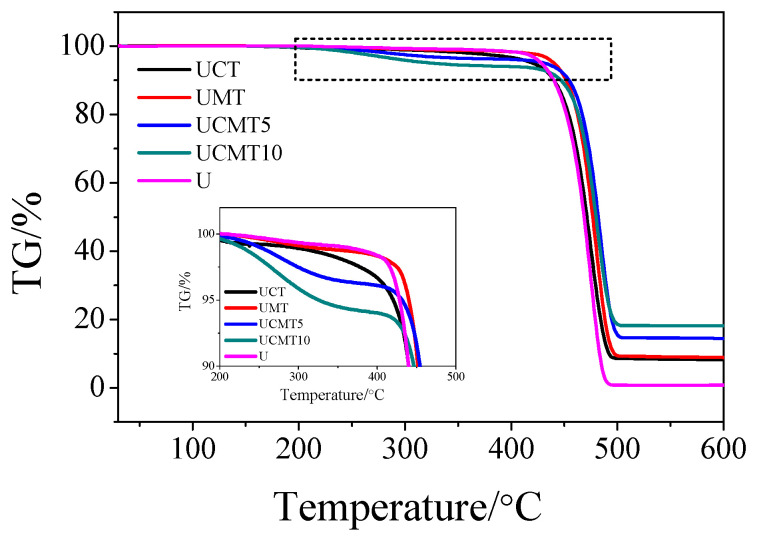
Thermogravimetry analysis of the UHMWPE matrix composite.

**Figure 3:**
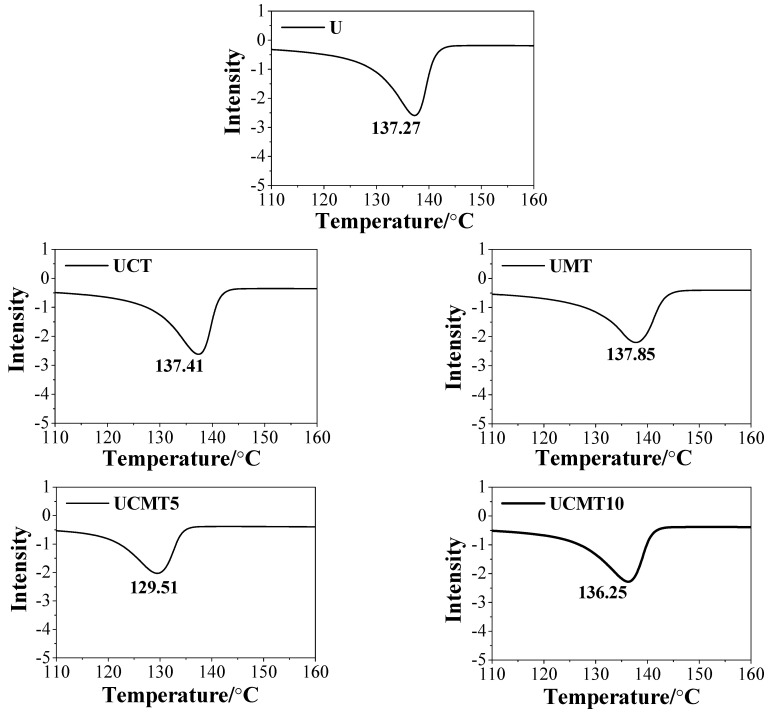
Differential scanning calorimetry analysis of the UHMWPE composites.

Figure 2 and Figure 3 present the TGA and DSC results of the UHMWPE composites when the aging time was 0 h. From Figure 2, we can see clearly that the decomposition temperatures of UCT, UCMT5, and UCMT10 decreased with an increase in the content of second particles as compared with that of pure UHMWPE materials, especially when the TiC content was 10%. According to DSC findings (Figure 3), the addition of ceramic particles decreased the melting temperatures for composites. This may be because the high content of second particles reduces the crystallinity of UHMWPE matrix composites as the second-phase particles occupy the free volume of the polymer material.

A tensile test was carried out to evaluate the mechanical properties of the UHMWPE matrix composites. The results are shown in Table 3. It is clear that the addition of MoS_2_, TiC, and CF significantly decreased the stress of the UHMWPE composites, which could have been due to the weak bonding between the inorganic filler and organic matrix. In addition, the stress of the composites was essentially maintained with an increasing aging time, which could be attributed to the oxidation resistance of the compacted UHMWPE matrix.

### 3.1. Friction and Wear Performance Test

**Figure 4:**
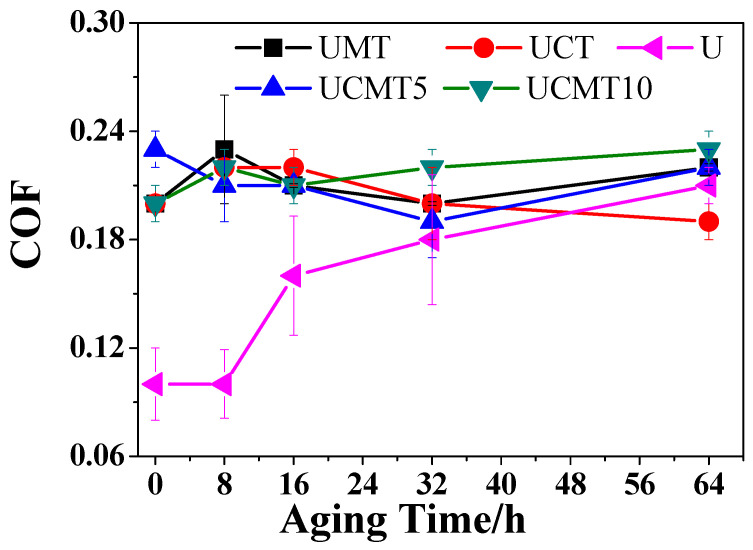
COFs of UHMWPE matrix composites at various aging times.

Figure 4 shows the changing trend of COFs vs. aging time as UHMWPE matrix composites coupled with a GCr15 bearing steel ball. The COFs initially decreased from 0 h to 16/32 h and then increased slightly with an increasing aging time of up to 64 h. Overall, all four composites exhibited excellent lubricating properties and the variation range was 0.19–0.23. For UMT and UCT composites, the COFs increased slightly with an increasing aging time from 0 to 8 h while these values reached the lowest value of 0.2 and 0.19 with increasing aging times of up to 32 and 64 h, respectively. For UCMT5 and UCMT10 composites, the fluctuation for COFs was slightly different: from 0 h to 32 h, the COFs gradually decreased and reached the lowest value of 0.19 at 32 h. With an increasing aging time up to 64 h, the COF increased continuously to the highest value of 0.22. This phenomenon can be explained through the following aspects: as the aging time increases from 0 h to 16/32 h, oxidative film formed on the worn surface is quickly removed and the fresh layer is exposed, so the COFs of these composites decrease while, as the aging time increases to 64 h, high shear force leads to an increase in the thickness of oxidative film, so the COFs increase to the maximum value.

As for the effect of second-particle content, the results showed that when the aging times were 0 h, 8 h, and 16 h, data were basically the same for all four composites, amounting to around 0.21. Meanwhile, when the aging time was 32 h, the UCMT5 composite exhibited the lowest COF and UCMT10 presented the highest value. At 64 h, the composite of UCT presented the best lubricating properties (0.19) while UMT, UCMT5, and UCMT10 composites showed similar values (around 0.22). Moreover, we can conclude that the synergistic lubricating effect of CF and TiC was better than that of MoS_2_ and TiC, especially when the aging time exceeded 32 h.

**Figure 5:**
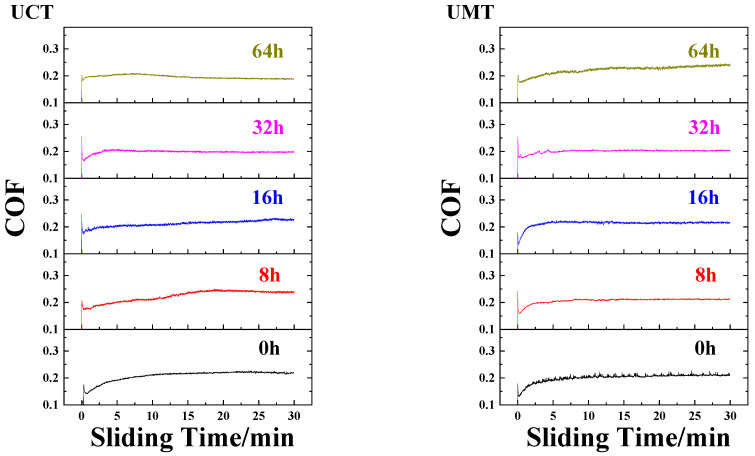

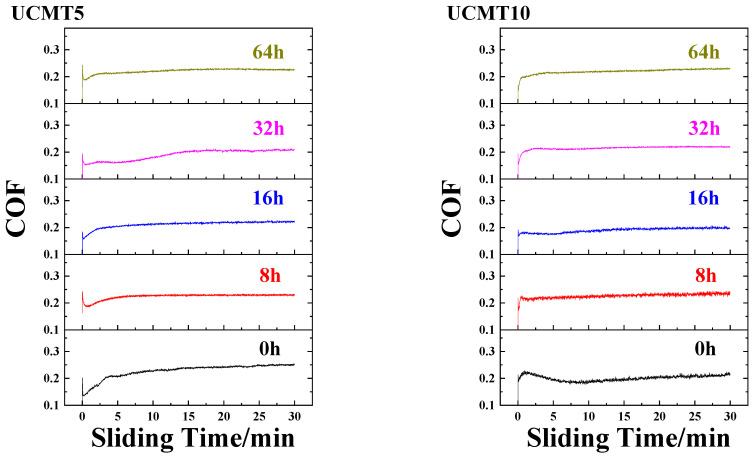
Typical friction curve of UHMWPE matrix composites at different aging times.

Furthermore, when we compare the typical curve of COFs versus sliding times at different aging times (Figure 5), we can see that the variations in the COF versus sliding time were small and stable for all four composites. Moreover, the running-in stages for all four composites were short (about five minutes) and the fluctuation in the values was at a slightly stable stage during the sliding process, indicating that a stable lubricating film had formed on the worn surface.

**Figure 6:**
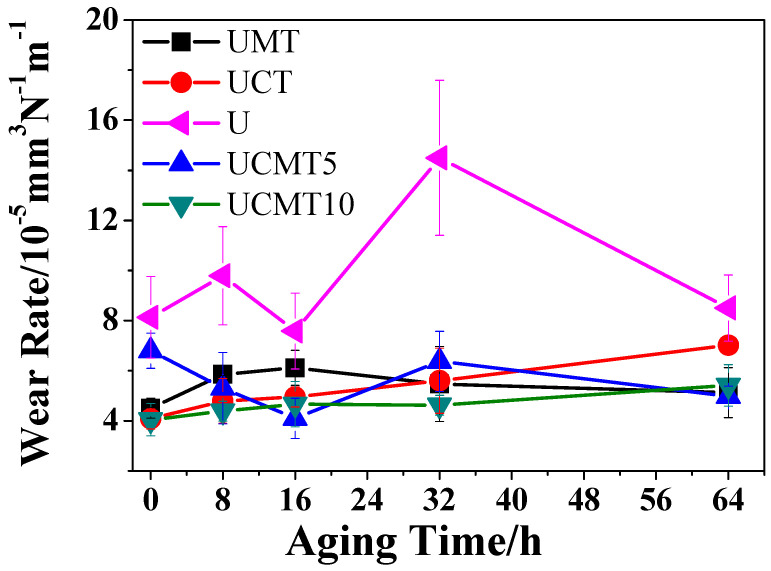
Wear rates of composites at different aging times.

The change in wear rates for UHMWPE matrix composites with aging time is shown in Figure 6. It can be seen that the wear rate for UCT and UCMT10 gradually increased with an increasing aging time from 0 to 64 h (UCT: 4.1–7.0 × 10^−5^ mm^3^/Nm, UPM10: 4.0–5.4 × 10^−5^ mm^3^/Nm). This can be attributed to the decreased hardness and strength of composites as the oxide layer on a worn surface increases with an increasing aging time, which accelerates fatigue wear and ultimately leads to an increase in the wear rate. In the case of the UCMT5 composite, the development of wear rate vs. aging time was different: from 0 to 16 h, the wear rate gradually decreased to the minimum value of around 4.1 × 10^−5^ mm^3^/Nm, then rose again slightly at 32 h and fell slightly with increasing aging of up to 64 h. For the UMT composite, the wear rate gradually increased to the maximum value when the aging time increased to 16 h (6.1 × 10^−5^ mm^3^/Nm), then continuously decreased to the relatively low value of 5.1 × 10^−5^ mm^3^/Nm at 64 h. We can attribute this to the reduced hardness and strength of composites as the friction heat on a worn surface increases at a high sliding speed, which accelerates the fatigue wear and eventually leads to an increase in the wear rate. Due to the synergistic reinforcement of CF, MoS_2_, and the ceramic particles (TiC), all four composites exhibited lower wear rates compared to pure UHMWPE (7.6–15.5 × 10^−5^ mm^3^/Nm), as reported in our previous work, especially at an aging time of up to 16 h [25]. In comparison, the synergistic lubricating effect of MoS_2_, CF, and TiC ceramics was superior to those of MoS_2_/TiC and CF/TiC.

**Figure 7:**
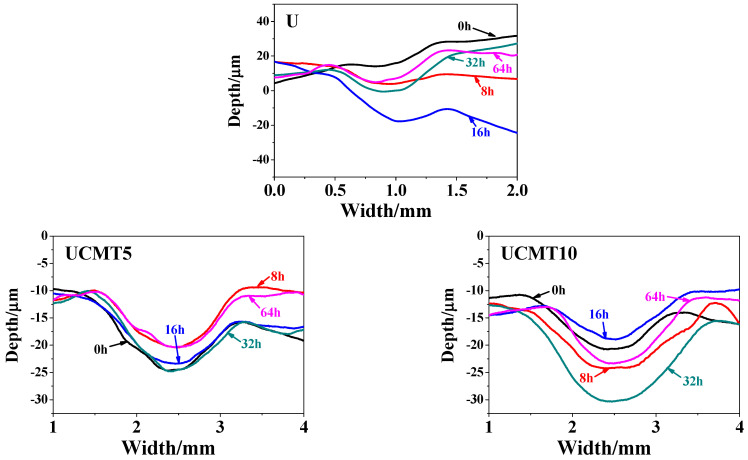
Profiles of the wear track for UCMT5 and UCMT10 at different aging times.

**Figure 8:**
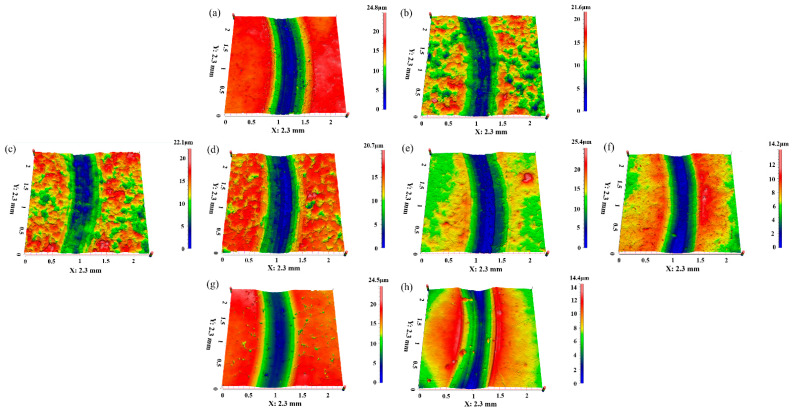
Three-dimensional morphologies of the worn surface for UHMWPE composites: (**a**) UCMT5—0 h, (**b**) UCMT5—8 h, (**c**) UCMT5—16 h (**d**) UCMT5—32 h, (**e**) UCMT5—64 h, (**f**) UCMT10—64 h; (**g**) UCT—64 h, (**h**) UMT—64 h.

In addition, the change in the wear rate could also be seen in the cross-section and three-dimensional morphology of the worn scar, as shown in Figure 7 and Figure 8. For UCMT5, the worn tracks were significantly wider and deeper at aging times of 0, 16, and 32 h (Figure 7 and Figure 8a,c,d) while they were shallow at 8 and 64 h (Figure 8b,e). For the other three composites, the worn surface was flat for UCMT10/UMT (Figure 8f,h) and deep for UCT (Figure 8g) at 64 h. Moreover, we could also see that the sliding surface was smooth and there was almost no wear debris for these composites when the aging time was 64 h (Figure 8e–h). As the aging time increased from 0 h to 64 h, it could be seen that the worn surface was smooth at 0 h, 32 h, and 64 h while the same flaking pits and delaminated layer appeared at 8 h and 16 h for the UCMT5 composite.

### 3.2. Worn Surface Analysis

**Figure 9:**
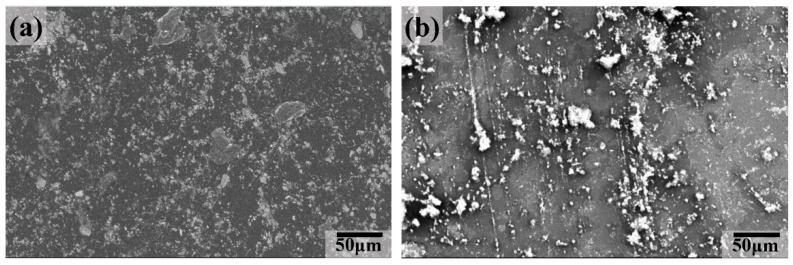

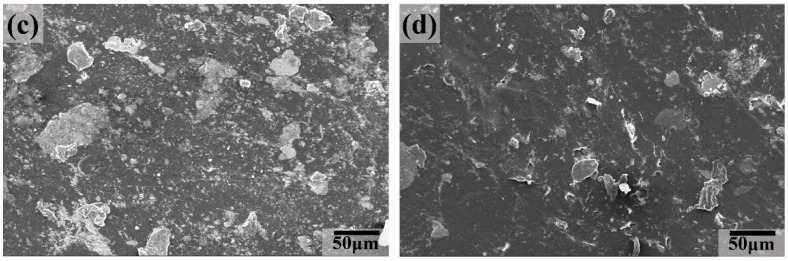
SEM images of the worn surface for UMT composite at different aging times: (**a**) 8 h, (**b**) 16 h, (**c**) 32 h, and (**d**) 64 h.

**Figure 10:**
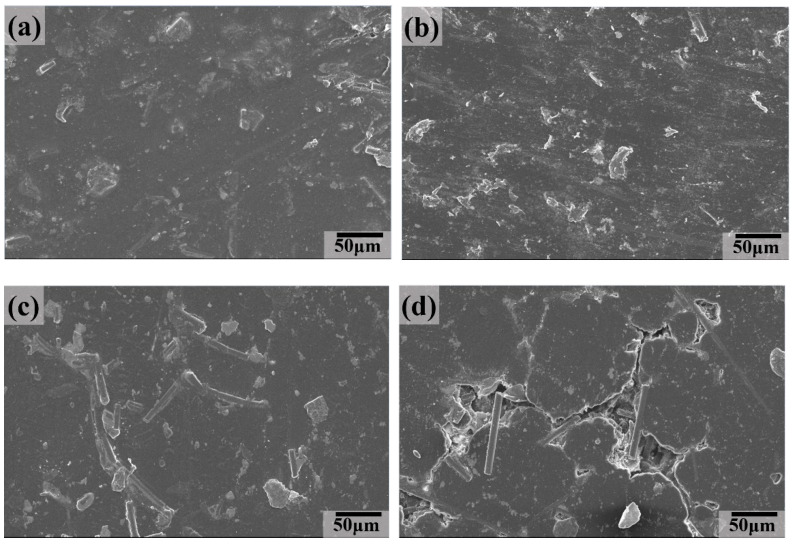
SEM images of the worn surface for UCT composite at different aging times: (**a**) 8 h, (**b**) 16 h, (**c**) 32 h, and (**d**) 64 h.

**Figure 11:**
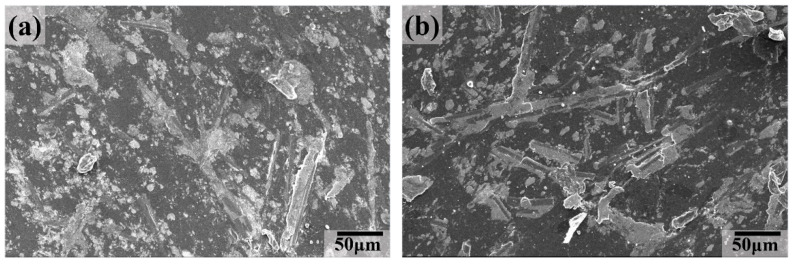

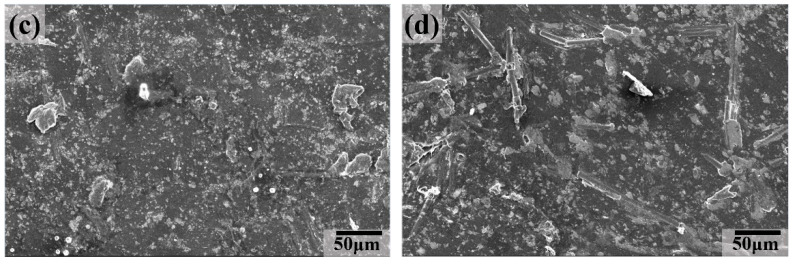
SEM images of the worn surface for UCMT5 composite at different aging times: (**a**) 8 h, (**b**) 16 h, (**c**) 32 h, and (**d**) 64 h.

**Figure 12:**
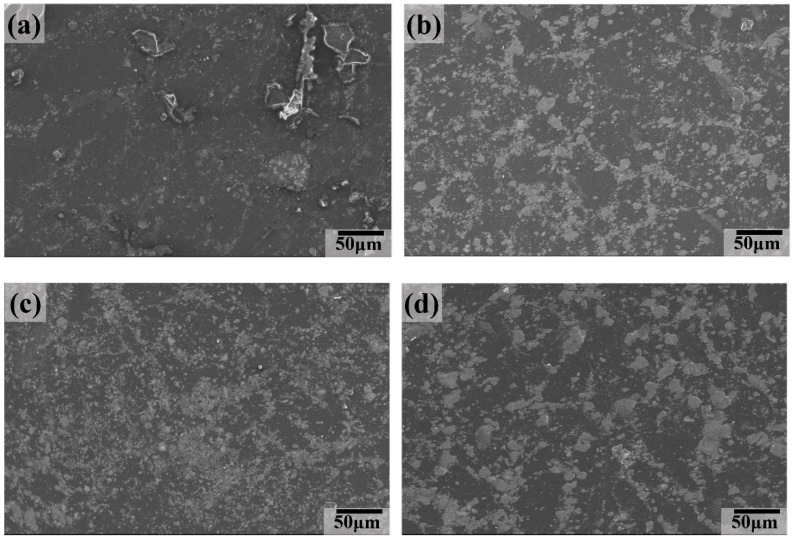
SEM images of the worn surface for UCMT10 composite at different aging times: (**a**) 8 h, (**b**) 16 h, (**c**) 32 h, and (**d**) 64 h.

To better understand the wear mechanism of the UHMWPE matrix composites, SEM examinations were performed to analyze the morphology of the worn surface (Figure 9, Figure 10, Figure 11 and Figure 12). Figure 9 shows the morphologies of the worn surface for the UMT composite after being coupled with a GCr15 stainless steel ball at different aging time. At 8 h, tiny furrows with some flaking pits constituted the main feature of the worn surface, indicating that adhesive wear was the main wear mechanism. Large wear debris except for some furrows appeared on the worn surface at 16 and 32 h, indicating that fatigue wear was the main wear mechanism. At 64 h, a smooth tribofilm with some small flaking pits appeared, indicating that the main wear mechanism was tiny abrasive wear.

The SEM images of the worn surface of the UCT composite at different aging times are shown in Figure 10. When the aging time was between 8 and 32 h, only a few tiny furrows instead of large flaking pits appeared on the worn surface compared to the UMT composite. It can also be clearly seen that CF peeled and smeared on the worn surface during the sliding process, which could have led to a relatively low COF and wear rate (Figure 3 and Figure 5). When the aging time increased to 64 h, the worn surface was covered with a large delamination and cracks, indicating that fatigue wear was the main wear mechanism.

Figure 11 illustrates the SEM images of the worn surface for the UCMT5 composite after thermal oxygen aging treatment at different aging times. The main features were similar to those of the UMT composite. Both were characterized by slight grooves and flaking pits; also, the carbon fibers had smeared on the sliding surface, indicating that abrasive wear was the main wear mechanism.

Figure 12 shows the SEM images of the worn surface of the UCMT10 composite after sliding against a GCr15 stainless steel ball at different aging times from 8 h to 64 h. It shows a smooth lubricating layer with some delaminated pits, indicating that the main wear mechanism was abrasive wear. Combined with the evolution of COFs (Figure 4) and the wear rate (Figure 6), we may deduce that a high content of ceramic particles decreased the lubricating properties while the wear resistance performance was improved.

**Figure 13:**
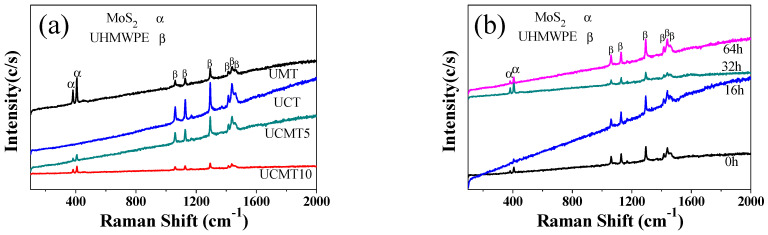
Raman spectrum for the worn surfaces of the composites at different aging times: (**a**) four composites at 64 h; (**b**) UCMT5 at 0 h, 16 h, 32 h, and 64 h.

As we all know, the friction and wear properties of composite materials are strongly dependent on the surface composition during the sliding process. To further analyze the influence of aging time and ceramic particle content on the friction and wear properties of composite materials, the Raman spectra of the worn surfaces of the composites at different aging times were investigated (see Figure 13). Compared with the other three composites, the UCT composite showed the highest intensity of the matrix peak at an aging time of 64 h (Figure 13a), indicating that the oxidative layers formed on the sliding surfaces of the composites UMT, UCMT5, and UCMT10 were thicker than that of UCT. As reported, the oxidative layer could increase the wear resistance but decrease the lubricating properties of these materials, which was consistent with the reported results showing that the MoS_2_ solid lubricant was easily oxidized and CF could improve the oxidative resistance properties of the composite in the dry sliding process [34], thus indicating the relatively low COF and high wear rate at 64 h of the UCT composite (Figure 3 and Figure 5). For the UCMT5 composite, we can find that the intensity of MoS_2_ peak increased from 0 to 32 h and decreased at 64 h. Based on these results, we may deduce that when the aging time was under 32 h, there was only some MoS_2_ smeared on the worn surface, while as the aging time increased to 64 h, the high content of the oxidative layer resulted in the wear debris peeling off from the matrix (Figure 7) and so exhibited a relatively high COF (Figure 3).

**Figure 14:**
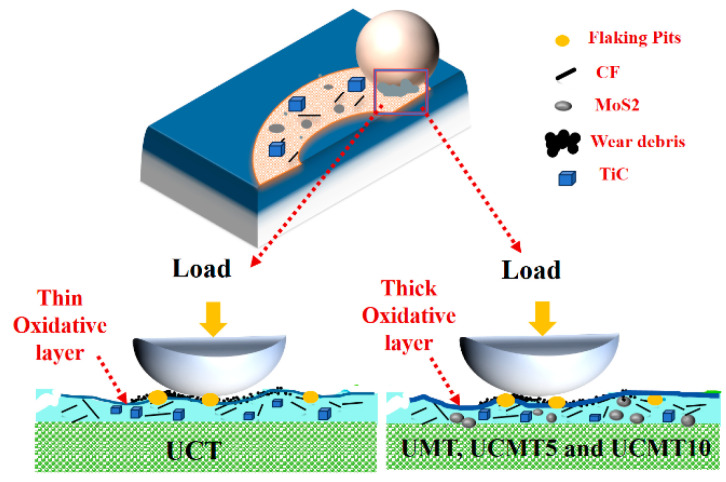
Schematic of wear mechanism for composites at 64 h.

A comprehensive comparison shows that the aging time and the ceramic content have significant influence on the tribological behavior of UHMWPE matrix composites. To better understand this behavior, a schematic diagram was drawn as shown in Figure 14. For UCT, the addition of CF increases the oxidative resistance of the composite and most of the oxidative film formed on the worn surface is removed during the sliding process so that the COF of the composite decreases to a minimum while the wear rate increases to the maximum value. For the UMT, UCMT5, and UCMT10 composites, the thickness of the oxidative film increases by 64 h due to the slight oxidation of MoS_2_ during the sliding process. In our previous work, we proved that the oxidative film formed on the worn surface could increase the wear resistance of the materials so that the COF increases and the wear rate decreases.

## 4. Conclusions

The UHMWPE matrix composites with CF, TiC, and MoS_2_ were prepared by the hot pressed sintering method and their frictional behaviors under different aging times (0–64 h) at 90 °C were investigated. The conclusions drawn from this work are as follows:

(1) The composites show excellent tribological performance at different aging times from 0 to 64 h. The COF and wear rate are approximately (0.19–0.23) and (4.09–7.01 × 10^−5^ mm^3^/Nm).
(2) In general, with an increase in the aging time, the COF in the mass decreases first and then increases to the maximum value. The wear rate in the mass increases slightly with an increase in the aging time.
(3) The low COF and wear properties for composites were attributed to the synergistic lubrication and reinforcement of TiC, MoS_2_, and CF. The SEM images presented that the main wear mechanisms were abrasive and fatigue wear.
(4) Regarding the difference in aging times, the testing temperature and surface structure changes during the sliding process also had important effects on the friction behavior of the composites. As a further investigation, it is believed that the transfer film and structural changes in the UHMWPE surface were the main reasons for the fluctuation of COFs and wear rates, so it will be interesting to investigate the influencing factors of transfer membrane formation.

## Figures and Tables

**Table 1:** Compositions of UHMWPE matrix composites.

Materials	Composition	UHMWPE (wt. %)	CF (wt. %)	MoS_2_ (wt. %)	TiC (wt. %)
U	UHMWPE	100	0	0	0
UMT	UHMWPE + MoS_2_ + TiC	90	0	5	5
UCT	UHMWPE + CF + TiC	90	5	0	5
UCMT5	UHMWPE + MoS_2_ + CF+TiC	85	5	5	5
UCMT10	UHMWPE + MoS_2_ + CF+TiC	80	5	5	10

**Table 2:** Tribotest conditions of UHMWPE matrix composites.

Material	UMT, UCT, UCMT5, UCMT10
Aging time	0 h, 8 h, 16 h, 32 h, 64 h
Sliding speed	637 rpm/0.33 ms^−1^
Load	10 N
Test time	30 min
Counterpart materials	GCr15

**Table 3:** Tensile properties of UHMWPE matrix composites.

Aging Time	0 h	8 h	16 h	32 h	64 h
Stress/MPa					
U	34 ± 1	33 ± 1	35 ± 1	36 ± 1	
UMT	28 ± 1	25 ± 3	21 ± 1	22 ± 4	26 ± 5
UCT	34 ± 1	38 ± 2	31 ± 1	39 ± 2	21 ± 2
UCMT5	25 ± 4	25 ± 4	24 ± 4	24 ± 1	21 ± 2
UCMT10	19 ± 1	20 ± 3	21 ± 3	21 ± 2	21 ± 3

## Data Availability

The original contributions presented in the study are included in the article, further inquiries can be directed to the corresponding author.

## References

[1] Duraccio D., Strongone V., Malucelli G., Auriemma F., De Rosa C., Mussano F., Genova T., Faga M. (2019). The role of alumina-zirconia loading on the mechanical and biological properties of UHMWPE for biomedical applications. Compos. Part B.

[2] Yang R., Chen X., Tian Y., Chen H., Boshkov N., Li H. (2021). An attempt to improve cavitation erosion resistance of UHMWPE coatings through enhancing thermal conductivity via the incorporation of copper frames. Surf. Coat. Technol..

[3] Hirwani J., Sinha S. (2023). Bio-tribological studies of structalit/UHMWPE composites as an alternative to UHMWPE for hip joint application. Wear.

[4] Shelly D., Lee S., Park S. (2024). Compatibilization of ultra-high molecular weight polyethylene (UHMWPE) fibers and their composites for superior mechanical performance: A concise review. Compos. Part B.

[5] Subhedar P., Padmanabhan D., Agrawal R., Singh G. A comprehensive review summarising the reinforcement of UHMWPE composite scaffolds as an artificial implant biomaterials. Mater. Today.

[6] Visco A., Brahimi S., Giudice F., Scolaro C., Sili A. (2024). Vitamin E effects on the wear resistance of UHMWPE sheets against an EBM-produced Ti6Al4V pin. Mater. Lett..

[7] Wu J., Yang T., Dong P., Zhang Q., Wang K. (2024). Simultaneously enhanced antioxidation and wear resistance in UHMWPE molded plastic by adding natural polyphenol antioxidant. Polymer.

[8] Xiao Y., Zhang L., Wei B., Lu C., Li L. (2024). Organic-inorganic core-shell materials as reinforced fillers of UHMWPE to improve its tribological and mechanical properties. Colloids Surf. A.

[9] Qin H., Zhou X., Zhao X., Xing J., Yan Z. (2015). A new rubber/UHMWPE alloy for water-lubricated stern bearings. Wear.

[10] Sharma S., Bijwe J., Panier S., Sharma M. (2015). Abrasive wear performance of SiC-UHMWPE nano-composites—Influence of amount and size. Wear.

[11] Sreekanth P., Kanagaraj S. (2015). Influence of multi walled carbon nanotubes reinforcement and gamma irradiation on the wear behaviour of UHMWPE. Wear.

[12] Yang G., Meng G., Gao H., Lin Q. (2022). Micromorphology and mechanical properties of UHMWPE/CNF composites under accelerated aging. Polym. Compos..

[13] Padhan M., Marathe U., Bijwe J. (2023). A comparative assessment of nano and microparticles of carbides for performance augmentation of UHMWPE in abrasive and erosive wear modes. Wear.

[14] Cai T., Zhan S., Yang T., Jia D., Tu J., Li Y., Li J., Duan H. (2023). Influence mechanism of organic-modified α-zirconium phosphate on tribological properties of UHMWPE. Wear.

[15] Meng Z., Wang Y., Liu H., Yan Y., Yan F. (2022). Reinforced UHMWPE composites by grafting TiO_2_ on ATP nanofibers for improving thermal and tribological properties. Tribol. Int..

[16] Uyor U., Popoola A., Popoola O. (2024). Enhancement of the tribological and thermal properties of UHMWPE based ternary nanocomposites containing graphene and titanium titride. J. Polym. Eng..

[17] Wu Y., Dong C., Bai X., Yuan C. (2023). Enhancing friction and vibration reduction properties of a polymer using h-BN particles. Wear.

[18] Lin Z., Zhang K., Ye J., Li X., Zhao X., Qu T., Liu Q., Gao B. (2022). The effects of filler type on the friction and wear performance of PEEK and PTFE composites under hybrid wear conditions. Wear.

[19] Song F., Yang Z., Zhao G., Wang Q., Zhang X., Wang T. (2017). Tribological performance of filled PTFE-based friction material for ultrasonic motor under different temperature and vacuum degrees. J. Appl. Polym. Sci..

[20] Xu J., Han T., Zhang C., Luo J. (2024). Superlubricating UHMWPE composites with functionalized carbon nanotubes reinforcement applicable to artificial joints. Tribol. Int..

[21] Gürgen S. (2019). Wear performance of UHMWPE based composites including nano-sized fumed silica. Compos. Part B.

[22] Gürgen S. (2022). Wear behavior of UHMWPE composites under oxidative effect. Polym. Degrad. Stab..

[23] Ruggiero A., D’Amato R., Gómez E. (2015). Experimental analysis of tribological behavior of UHMWPE against AISI420C and against TiAl6V4 alloy under dry and lubricated conditions. Tribol. Int..

[24] Zhen J., Zhu S., Cheng J., Qiao Z., Liu W., Yang J. (2016). Effects of sliding speed and testing temperature on the tribological behavior of a nickel-alloy based solid-lubricating composite. Wear.

[25] Zhen J., Han Y., Zhu L., Hou W., Liu Y., Huang W., Yang L., Yuan L., Jia Z., Zhang R. (2023). MoS_2_/CF synergistic enhancement to improve the friction and wear properties of UHMWPE composites. Tribol. Int..

[26] Gangwani P., Kovač J., Emami N., Kalin M. (2024). Effect of multi-scale fillers on the tribological behavior of UHMWPE composites in water-lubricated contacts. Tribol. Int..

[27] Lin Y., Guo Z., Yuan C. (2022). Effects of solid lubricants on the tribological behavior of steel-backed UHMWPE fabric composites. J. Appl. Polym. Sci..

[28] Yang Z., Guo Z., Yang Z., Wang C., Yuan C. (2021). Study on tribological properties of a novel composite by filling microcapsules into UHMWPE matrix for water lubrication. Tribol. Int..

[29] Zhou S., Zhang Q., Wu C., Huang J. (2013). Effect of carbon fiber reinforcement on the mechanical and tribological properties of polyamide6/polyphenylene sulfide composites. Mater. Des..

[30] Rahman M., Biswas M., Hoque K. (2022). Recent development on micro-texturing of UHMWPE surfaces for orthopedic bearings: A review. Biotribology.

[31] Cheng B., Shang H., Duan H., Chen Q., Li J., Shao T. (2024). Influence of laser-induced surface carbonization on the tribological properties of UHMWPE in a seawater environment. Appl. Surf. Sci..

[32] Menezes P., Kailas S. (2016). Role of surface texture and roughness parameters on friction and transfer film formation when UHMWPE sliding against steel. Biosurface Biotribology.

[33] Montes S., Dominguez L., Barceinas S. (2021). Effect of surface texturing of UHMWPE on the coefficient of friction under arthrokinematic and loading conditions corresponding to the walking cycle. Mater. Lett..

[34] Chen B., Li X., Jia Y., Li X., Yang J., Yan F., Li C. (2018). MoS_2_ nanosheets-decorated carbon fiber hybrid for improving the friction and wear properties of polyimide composite. Compos. Part A.

